# Supplementary material for: Rab35 governs apicobasal polarity through regulation of actin dynamics during sprouting angiogenesis
Source: Nat Commun. 2022 Sep 8;13:5276. doi: 10.1038/s41467-022-32853-5 (PMC9458672; doi:10.1038/s41467-022-32853-5)
Supplement: Supplementary file 1 — Supplementary Information [file 41467_2022_32853_MOESM1_ESM.pdf]

Supplemental Information: Rab35 Governs Apicobasal Polarity Through Regulation of Actin Dynamics During Sprouting Angiogenesis

Caitlin R. Francis[1], Hayle Kincross[1], and Erich J. Kushner[1]*

[1]Department of Biological Sciences, University of Denver, Denver, CO; *Author for correspondence:

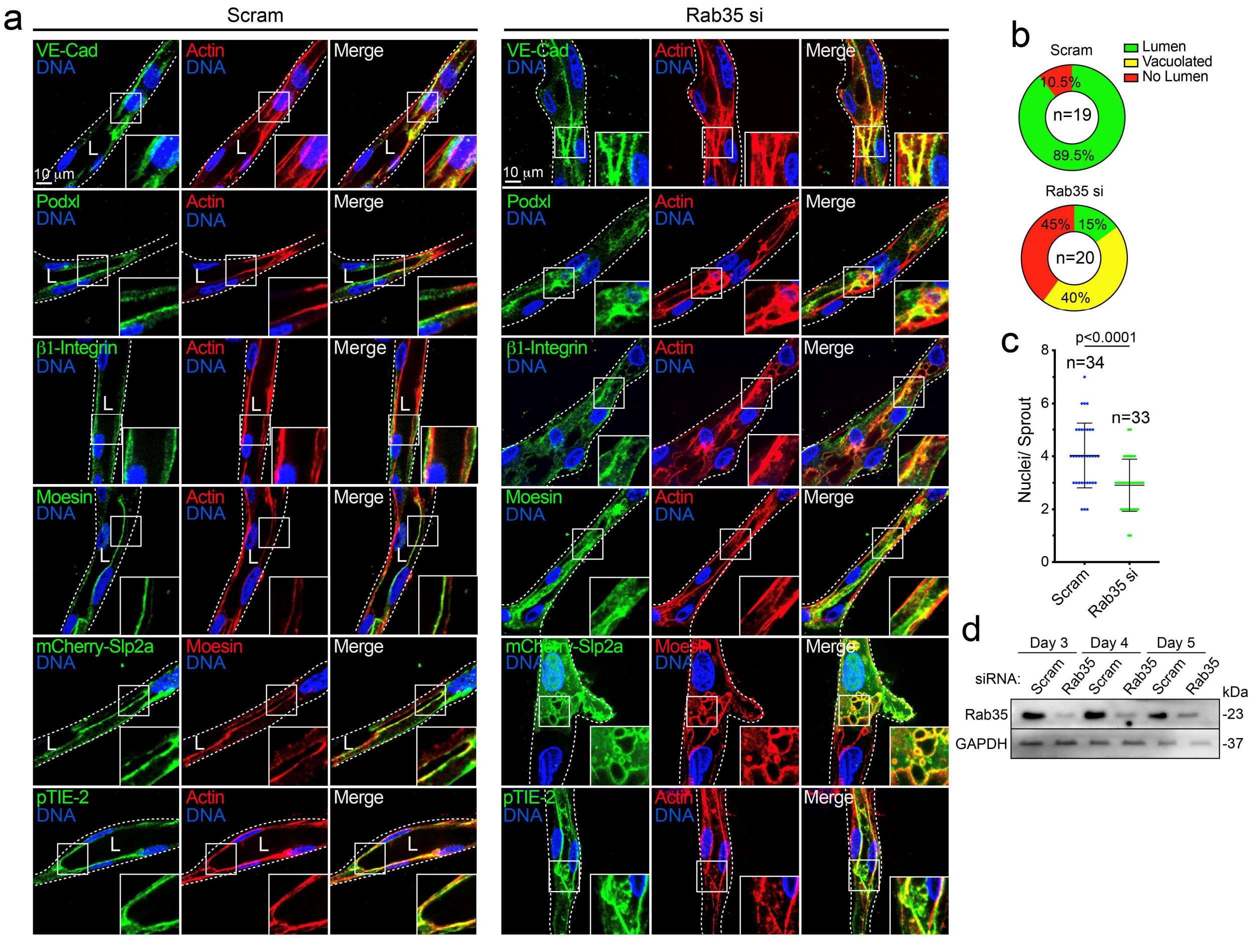

Supplemental Figure 1. Knockdown of Rab35 distorts cell apicobasal polarity. (**A**) Scramble (Scram) and Rab35 siRNA(si)-treated sprouts stained for VE-cadherin (VE-cad), podocalyxin (Podxl), β1-integrin, moesin or phosphorylated Tie2 (pTie2) apical and basal protein markers. Apical marker synaptotagmin-like protein 2a (mCherry-Slp2a) was transduced into sprouts. L denotes lumen and white dotted lines outline sprout exterior. (**B**) Quantification of lumen formation in Scram and Rab35 siRNA-treated sprouts. Lumens were defined as an open continuous cavity. Vacuolated sprouts were defined as sprouts lacking a contiguous lumen, while exhibiting an excess of large vacuoles. The no lumen group was defined as sprouts that had no visible cavity or vacuoles. n=number of sprouts. (**C**) Quantification of nuclei per sprout in Scram and Rab35 siRNA treated sprouts. n=number of sprouts. Error bars represent standard deviation, middle bars are the mean. **(D)** Western blot of Rab35 knockdown (KD) cells lysed 3 days, 4 days, and 5 days post siRNA treatment. Statistical significance was assessed with an unpaired t-test or a 1-way

ANOVA followed by a Dunnett multiple comparisons test. Insets are areas of higher magnification. All experiments were done using human umbilical vein endothelial cells in triplicate.

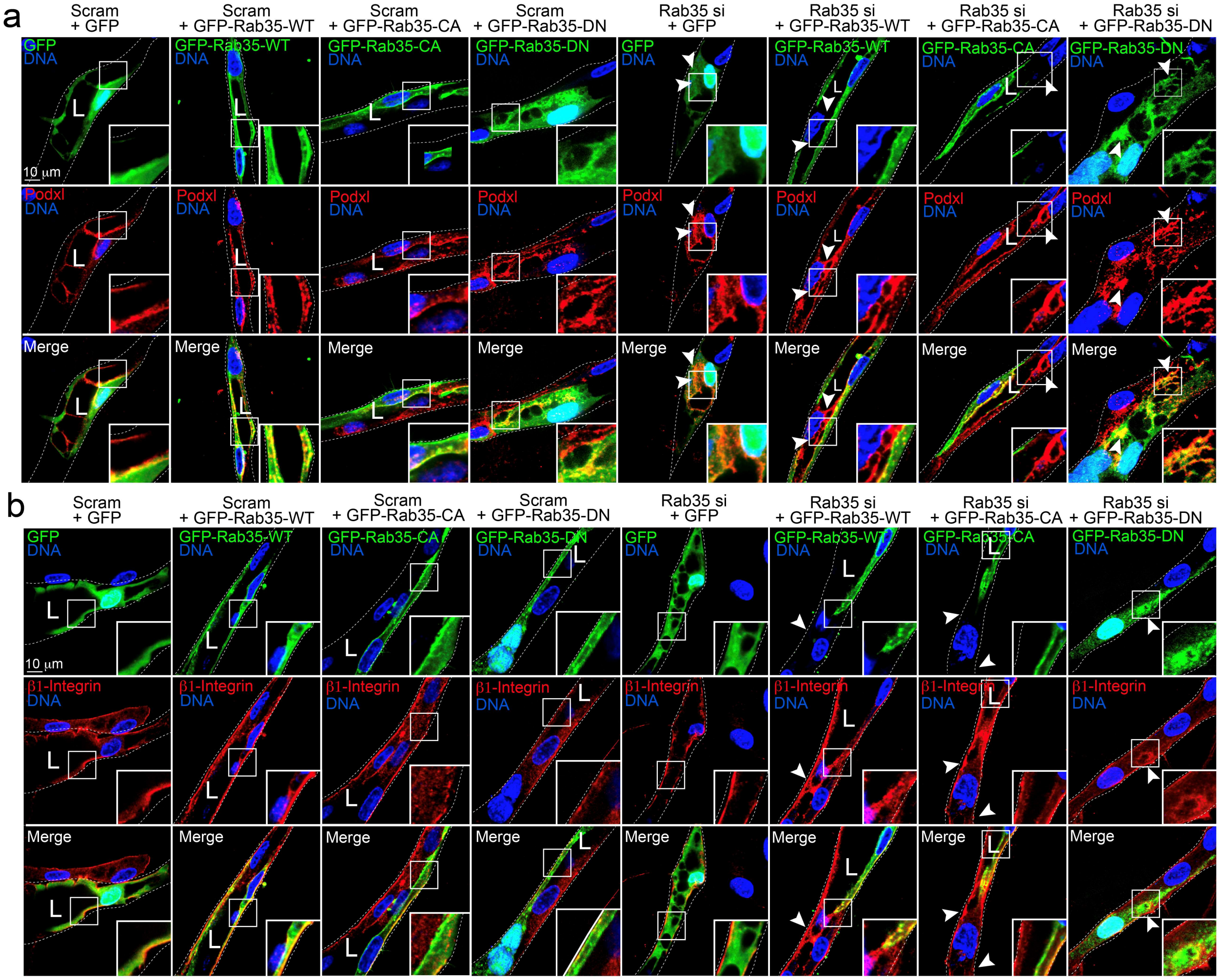

Supplemental Figure 2. Rab35 knockdown disrupts sprout polarity programs. (**A,B**) Representative images of Scram and Rab35 siRNA (si) knockdown (KD) sprouts transfected with GFP or GFP-Rab35 wild-type (WT), constitutively-active (CA) or dominant-negative (DN) for rescues. Sprouts were also stained for apical marker podocalyxin (Podxl) or basal marker β1-integrin. Arrowheads denote abnormal localization of podocalyxin or β1-integrin. L denotes lumen in all images. White dotted lines mark sprout exterior. Insets are areas of higher magnification. All experiments were done using human umbilical vein endothelial cells in triplicate.

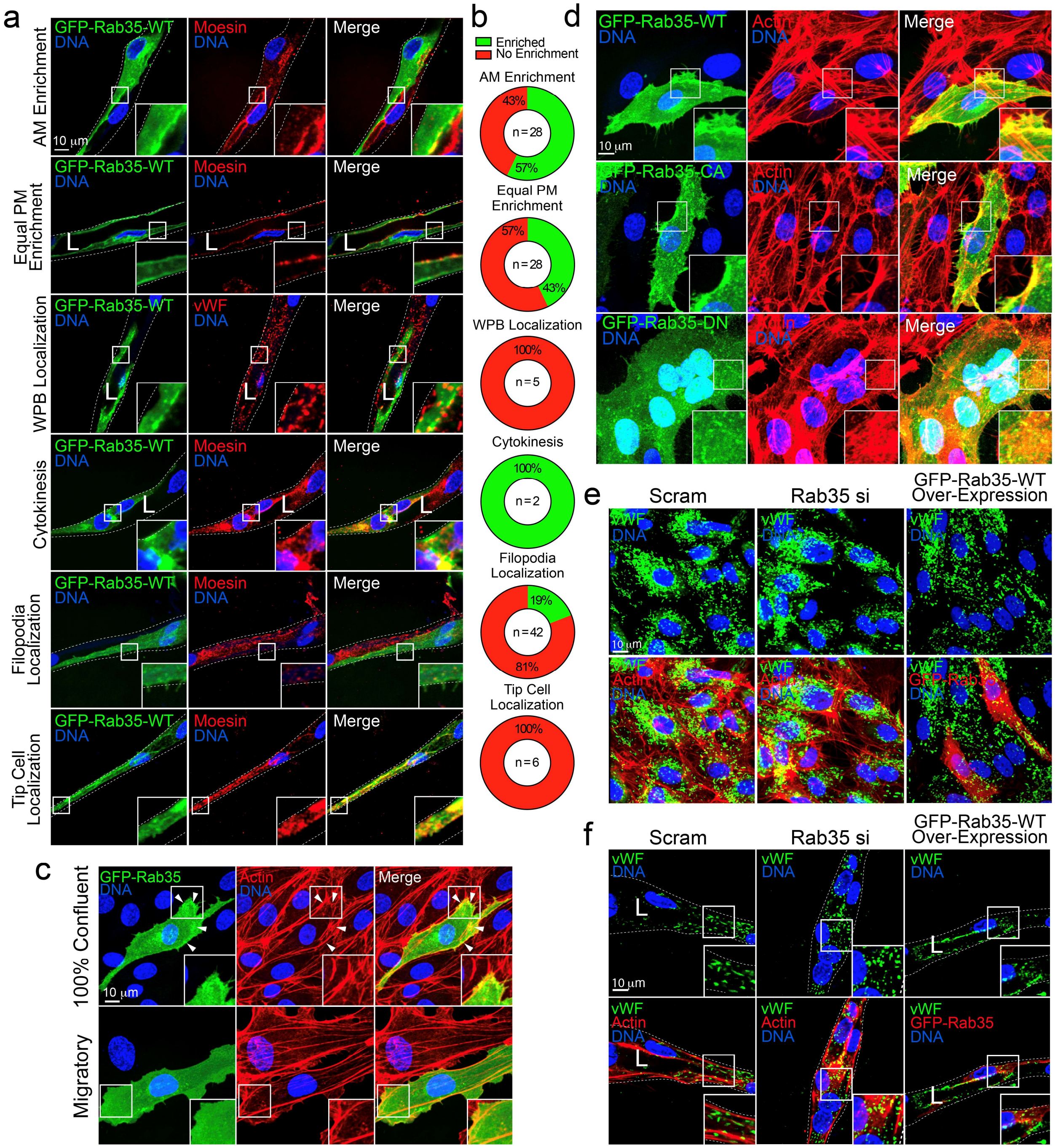

Supplemental Figure 3. Rab35 localizes to the plasma membrane and not to Weibel-Palade Bodies. (**A**) Representative images of GFP-Rab35-wild-type (WT) localization binned by its proximity to the apical plasma membrane (AM), equal enrichment at the basal and apical plasma membrane (equal plasma membrane (PM) enrichment), Weibel-Palade bodies (WPBs), at sites of cytokinesis, filopodia, and most distal cell in the sprout (tip cell). Sprouts were stained for moesin to mark the apical membrane. (**B**) Quantification of GFP-Rab35-WT enrichment with respect to the described conditions in panel A. (**C**) Representative images of GFP-Rab35 localization in 2-dimensional culture stain for actin. The top panels are of a confluent monolayer and the bottom panels are of migratory sub-confluent cells. Arrowheads indicate colocalization of

actin and GFP-Rab35. (**D**) Representative images of 2-dimensional localization of GFP-Rab35-WT (top panels), constitutively-active (CA, middle panels), and dominant-negative (DN, bottom panels) stained for actin. (**E**) Representative images of cells treated with scramble (Scram) or Rab35 siRNA (si) and stained for WPB marker von Willebrand Factor (vWF) and actin or overexpressing GFP-Rab35-WT. (**F**) Representative images of sprouts treated with Scram or Rab35 siRNA stained for vWF and actin or expressing GFP-Rab35-WT in sprouts. Insets are areas of higher magnification. White dotted lines mark sprout exterior. All experiments were done using human umbilical vein endothelial cells in triplicate.

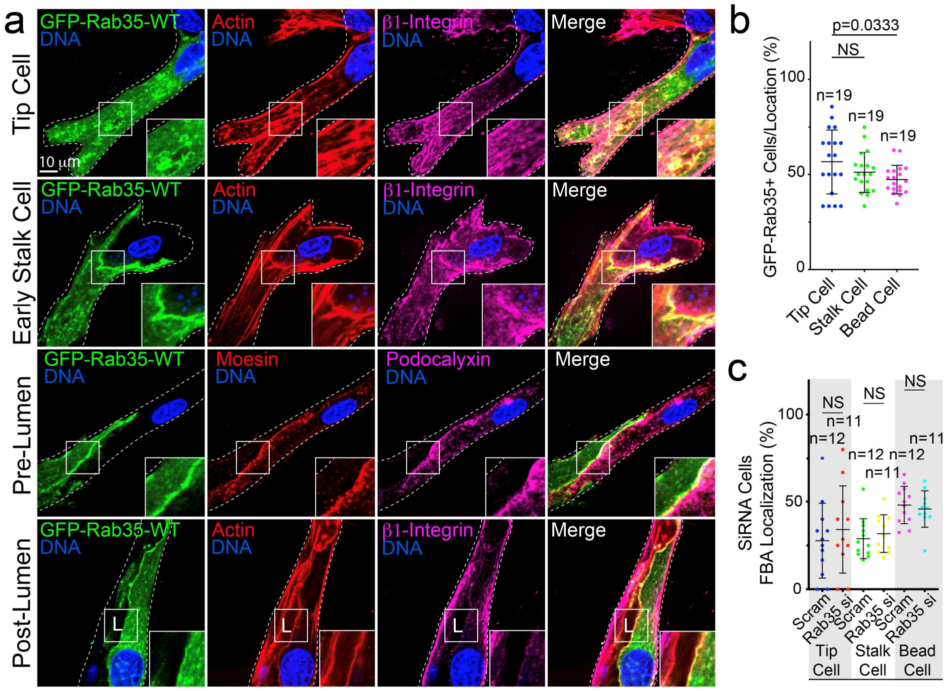

Supplemental Figure 4. Rab35 knockdown does not distort cell positioning in sprouts. (A) Representative image of GFP-Rab35-wild-type (WT) expression in the described sprout locations. **(B)** Quantification of GFP-Rab35-WT mosaic expression in the described sprout locations. n=number of cells. Error bars represent standard deviation, middle bars are the mean. **(C)** Quantification of siRNA (si)-treated cells marked with cell tracker binned by sprout location. n=number of cells. Error bars represent standard deviation, middle bars are the mean. NS=non-significant. Statistical significance was assessed with an unpaired t-test or a 1-way ANOVA followed by a Dunnett multiple comparisons test. Insets are areas of higher magnification. All experiments were done using human umbilical vein endothelial cells in triplicate.

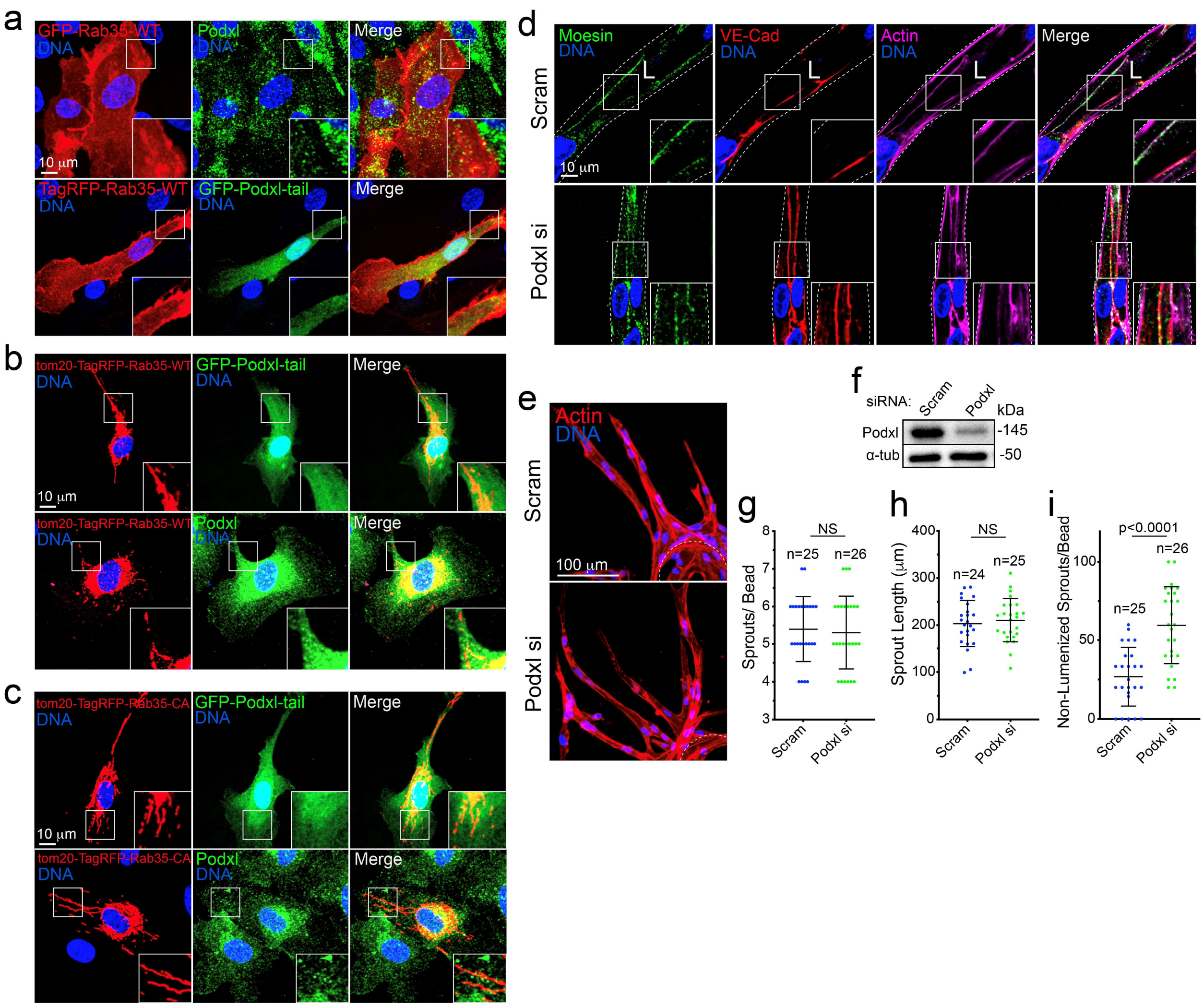

Supplemental Figure 5. Rab35 does not affect podocalyxin trafficking. (**A**) Two-dimensional localization of GFP-Rab35-wild-type (WT) stained for podocalyxin (Podxl) (top panels) or expressing of GFP-Podxl-tail (bottom panels). (**B**). Top panels- cell co-expressing tom20-TagRFP-Rab35-WT with GFP-Podxl-tail. Bottom panels- cell expressing tom20-TagRFP-Rab35-WT stained for endogenous podocalyxin. (**C**) Representative image of a cell co-expressing tom20-TagRFP-Rab35-constitutively active (CA) mutant with GFP-Podxl-tail. Bottom panels show a cell expressing tom20-tagRFP-Rab35-CA mutant stained for endogenous podocalyxin. (**D**) Representative image of sprouts treated with scramble (Scram) or podocalyxin siRNA (si) and stained for moesin, VE-cadherin (VE-cad) and actin. L denotes lumen. White dotted lines mark sprout exterior. (**E**) Sprout morphology for the same conditions as D. (**F**) Confirmation of siRNA-mediated knockdown by western blot. (**G-I**) Quantification of indicated sprouting parameters across groups. n=number of sprouts. Error bars represent standard deviation, middle bars are the mean. NS=non-significant. Statistical significance was assessed with an unpaired t-test or a 1-way ANOVA followed by a Dunnett multiple comparisons test. Insets are areas of higher magnification. All experiments were done using human umbilical vein endothelial cells in triplicate.

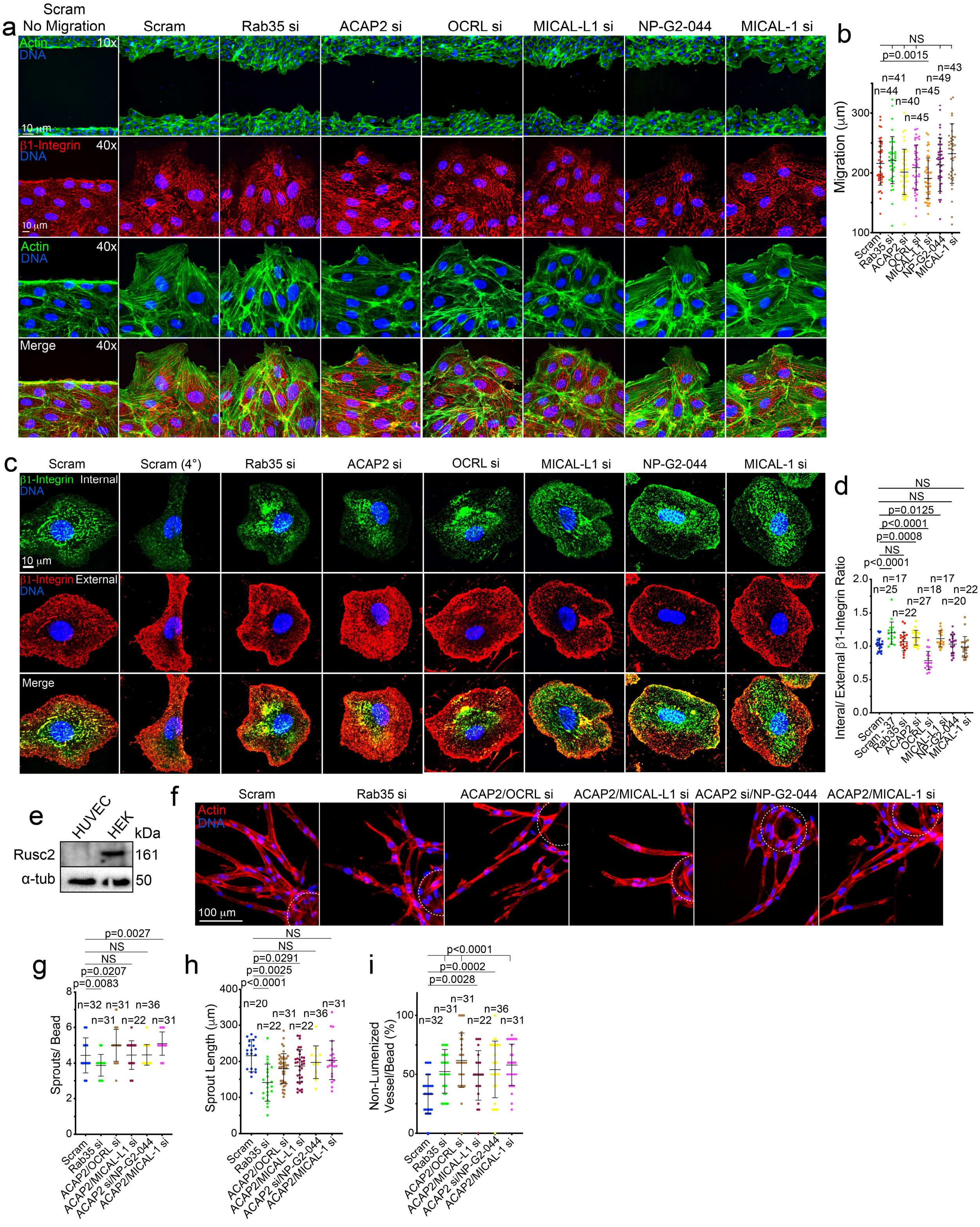

Supplemental Figure 6. Knockdown of Rab35 impacts integrin internalization, but not cell migration. (**A**) Migration assay in cells treated with scramble (Scram), Rab35, ACAP2, OCRL, MICAL-L1, or MICAL-1 siRNA (si) or Fascin inhibitor NP-G2-044. Cells were stained for β1-

integrin and actin. (**B**) Quantification for the migration assay in A. n=number of measurements. Error bars represent standard deviation, middle bars are the mean. (**C**) Antibody feeding assay to test for β1-integrin turnover between conditions. Cells were treated with indicated siRNA. Green channel represents internalized integrins, while the red channel marks only external integrins. As a control to inhibit endocytosis a group was held at 4°C. (**D**) Fluorescence intensity ratio of internalized to external β1-integrin in panel C**.** n=number of cells. Error bars represent standard deviation, middle bars are the mean. **(E)** Western blot image probing for Rusc2 in both HEK293 cells and human umbilical vein endothelial cells (HUVECs). **(F)** Representative images of sprout morphology between indicated groups. Dashed lines outline microbeads. **(G-I)** Graphs of indicated sprout parameters between groups. n= number of sprouts. Error bars represent standard deviation, middle bars are the mean. NS=non-significant. Statistical significance was assessed with an unpaired t-test or a 1-way ANOVA followed by a Dunnett multiple comparisons test. All experiments were done using Human umbilical vein endothelial cells in triplicate.

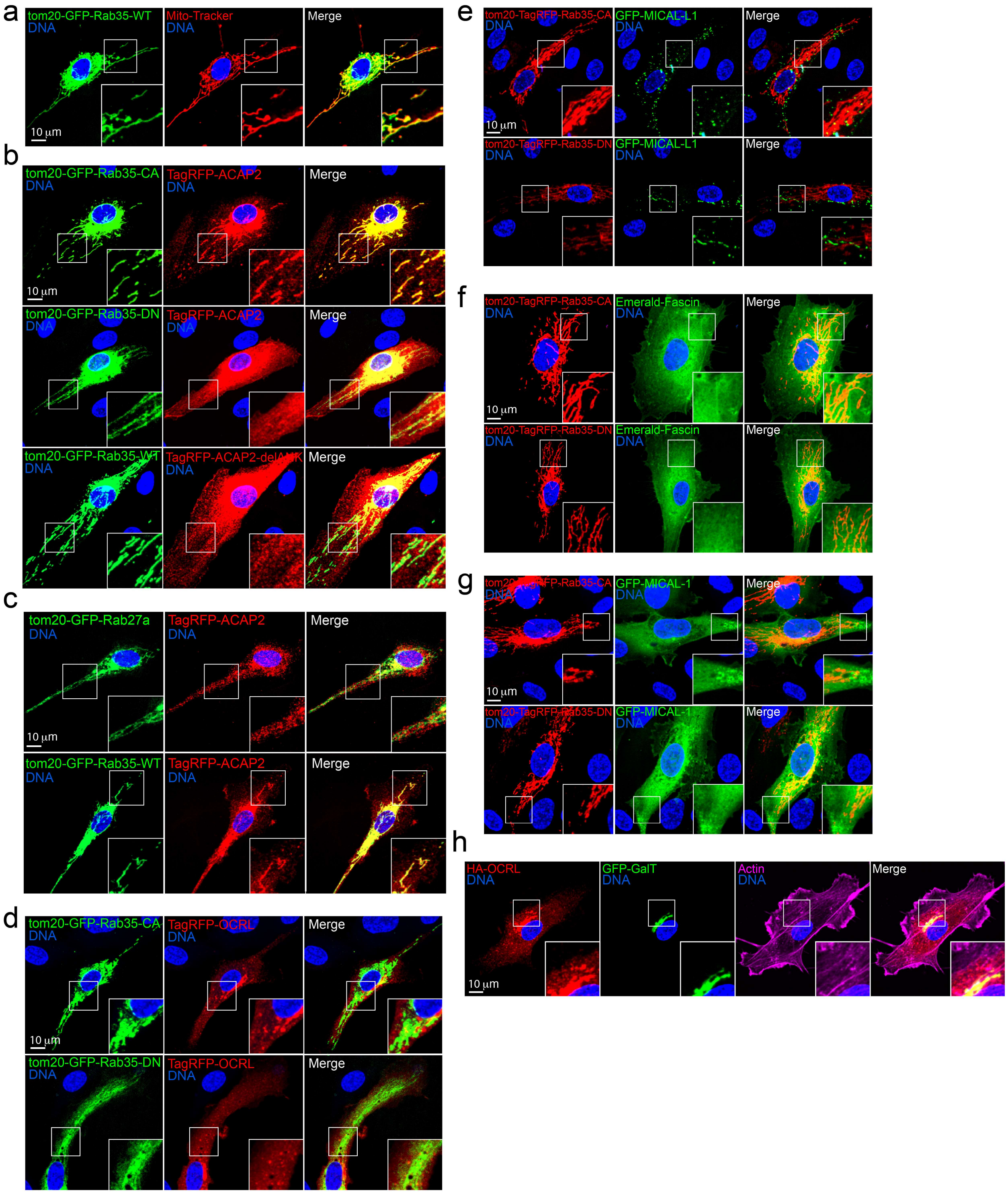

Supplemental Figure 7. Rab35 binds only ACAP2. (**A**) Cells stained for mitochondria (Mito-tracker) and transfected with tom20-tagRFP-Rab35-wild-type (WT). (**B**) Representative images of a cell co-expressing tom20-tagRFP-Rab35-WT, constitutively-active (CA), or dominant-negative (DN) variants with TagRFP-ACAP2 or ACAP2 with deleted ankyrin repeat domain

(delANK). (**C**) Representative image of a cell expressing tagRFP-ACAP2 and tom20-GFP-Rab27a-WT (top panels). Bottom panel is a representative image of a cell expressing of tom20-GFP-Rab35-WT with tagRFP-ACAP2. (**D**) Top panels- representative image of a cell expressing tom20-GFP-Rab35-CA and OCRL. Bottom panels- cell expressing tom20-GFP-Rab35-DN and TagRFP-OCRL. (**E**) Top panels- representative image of a cell expressing tom20-TagRFP-Rab35-CA and GFP-MICAL-L1. Bottom panels- cell expressing tom20-TagRFP-Rab35-DN and GFP-MICAL-L1. **(F)** Top panels- representative image of a cell expressing tom20-TagRFP-Rab35-CA and Emerald-Fascin. Bottom panels- cell expressing tom20-TagRFP-Rab35-DN and Emerald-Fascin. **(G)** Top panels- representative image of a cell expressing tom20-TagRFP-Rab35-CA and GFP-MICAL-1. Bottom panels- cell expressing tom20-TagRFP-Rab35-DN and GFP-MICAL-1. **(H)** Representative image of HA-OCRL and GFP-GalT (Golgi marker) localization. Insets are areas of higher magnification. All experiments were done using human umbilical vein endothelial cells in triplicate.

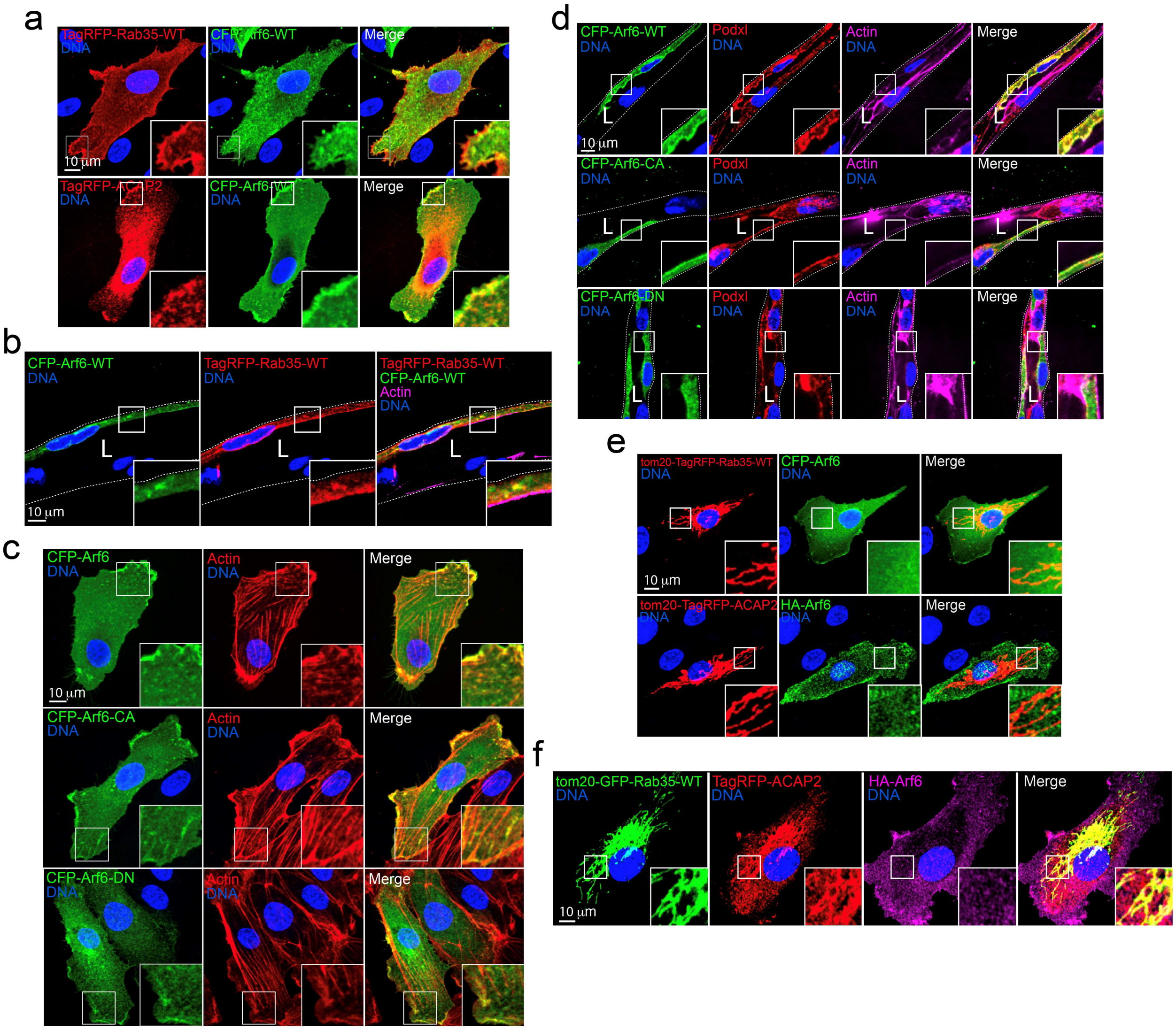

Supplemental Figure 8. Arf6 localization and interactions with Rab35 and ACAP2. (**A**) Two-dimensional localization of CFP-Arf6 with tagRFP-Rab35-wild-type (WT) (top panels) or tagRFP-ACAP2 (bottom panels). (**B**) Localization of tag-RFP-Rab35-WT and CFP-Arf6 in a sprout. **(C)** Two-dimensional localization of CFP-Arf6-WT (top panels), constitutively-active (CA, middle panels), or dominant-negative (DN, bottom panels) stained for actin. **(D)** Representative images of sprouts transduced with WT, CA, or DN CFP-Arf6 stained for podocalyxin (Podxl) and actin. (**E**) Top panel- representative image of a cell expressing tom20-tagRFP-Rab35-WT and CFP-Arf6. Bottom panel- representative image of a cell expressing tom20-tagRFP-ACAP2 and HA-Arf6-WT. **(F)** Representative image of a cell expressing tom20-GFP-Rab35-WT, tagRFP-ACAP2 and HA-Arf6-WT. L denotes lumen in all images. White dotted lines mark sprout exterior. Insets are areas of higher magnification. All experiments were done using human umbilical vein endothelial cells in triplicate.

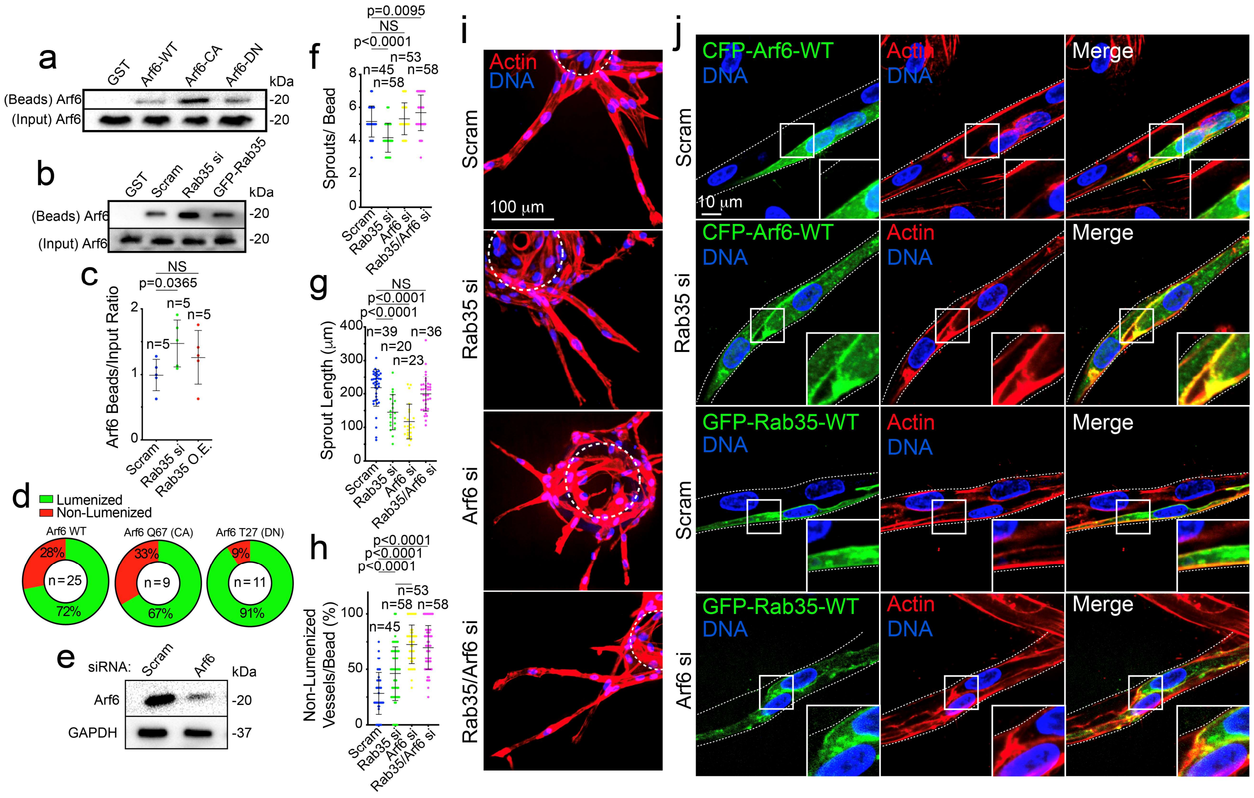

Supplemental Figure 9. Loss of Rab35 affects Arf6 activity. (**A**) Pulldown assay using GGA3 to probe for activated Arf6. Cells were transfected with wild-type (WT), constitutively-active (CA), or dominant-negative (DN) Arf6. (**B**) Pulldown assay using GGA3 to probe for activated Arf6. Cells were treated with scramble (Scram) or Rab35 siRNA (si) or transduced with GFP-Rab35-WT. **(C)** Quantification of Arf6 activity. n=number of pull-downs. Error bars represent standard deviation, middle bars are the mean. **(D)** Quantification of open or collapsed lumens after transduction with WT, CA, or DN CFP-Arf6. n= number of sprouts. **(E)** Western blot confirmation of siRNA knockdown (KD) of Arf6 (average 60.8% KD relative to control, n=3). **(F-H)** Graphs of indicated sprout parameters between groups. n=number of sprouts. Error bars represent standard deviation, middle bars are the mean. **(I)** Representative images of sprout morphology between indicated groups. Dashed lines outline microbeads. (**J**) Epistasis experiment showing CFP-Arf6-WT localization in Scram or Rab35 siRNA sprouts (top 2 panels) as well as GFP-Rab35-WT localization in Scram and Arf6 siRNA sprouts (bottom two panels). L denotes lumen in all images. White dotted lines mark sprout exterior. Insets are areas of higher magnification. NS=non-significant. Statistical significance was assessed with an unpaired t-test or a 1-way ANOVA followed by a Dunnett multiple comparisons test. All experiments were done using human umbilical vein endothelial cells in triplicate.

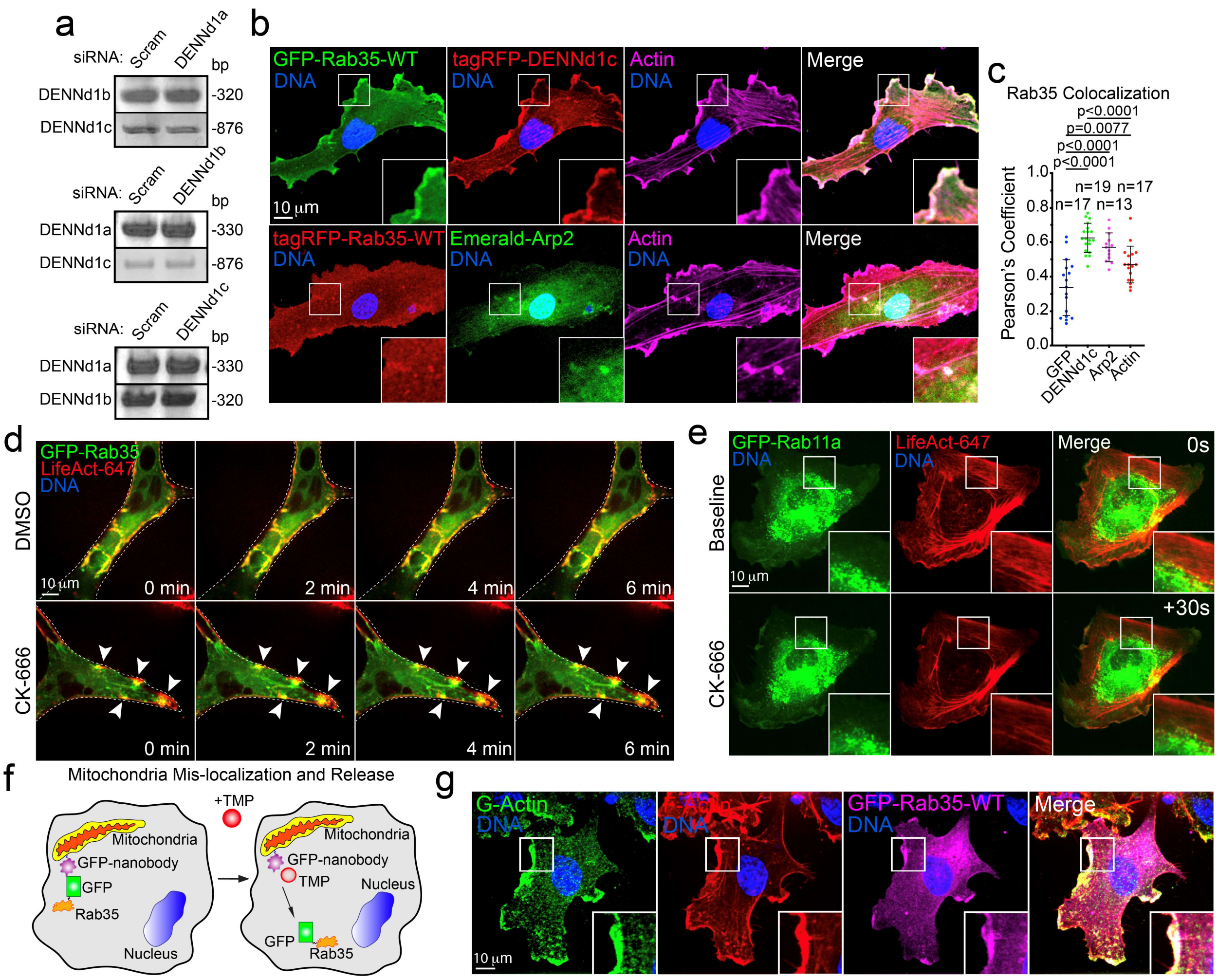

Supplemental Figure 10. Rab35 is recruited to sites of actin polymerization. (**A**) RT-PCR of DENNd1a-c knockdown. DENNd1's were individually knocked down using siRNA (si) and expression of the remaining two DENND1s were probed to test for compensation effects. Base-pair (BP). **(B)** Top panel- representative image of a cell expressing GFP-Rab35-wild-type (WT) and tagRFP-DENNd1c. Bottom panel- representative image of a cell expressing GFP-Rab35-WT and Emerald-Arp2. **(C)** Pearson's coefficient of Rab35 co-localization with described proteins. n=number of cells. Error bars represent standard deviation, middle bars are the mean. **(D)** GFP-Rab35-WT and LifeAct-tagRFP647 (647) co-expression in sprouts live-imaged with vehicle or following treatment with CK-666. Arrowheads indicate accumulations of GFP-Rab35-WT and LifeAct-647. Dotted line indicates sprout exterior. **(E)** Representative live-image of a cell expressing GFP-Rab11a and LifeAct-647 before and after CK-666 treatment. **(F)** Cartoon of a mitochondria-localized GFP-nanobody and controlled release of GFP-Rab35 upon treatment with Trimethoprim (TMP). In the absence of TMP the nanobody sequesters GFP or GFP-tagged

proteins. In the presence of TMP the GFP cargo is released. **(G)** Representative image of a cell expressing GFP-Rab35-WT and stained for filamentous (*F*) and globular (*G*) actin. Insets are areas of higher magnification. Statistical significance was assessed with an unpaired t-test or a 1-way ANOVA followed by a Dunnett multiple comparisons test. All experiments were done using human umbilical vein endothelial cells in triplicate.

A

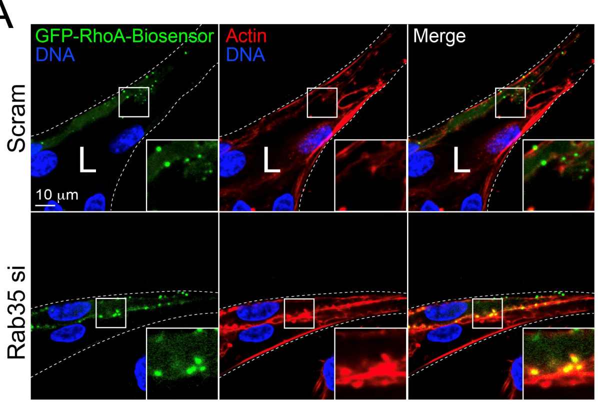

B

CRISPR Off-Target Gene Identification

Target	*Target Sequence*	*Mismatch*	*Number of Found Targets*	*Exonic Targets*	*Intronic Targets*
**Rab35-A**	CTTGAAGTCCACACCGAT	1	1	0	1
**Rab35-B**	CTTGAAGTCGACTCCTATA	1	1	0	1
**Rab35-A**	CTTGAAGTCCACACCGAT	2	4	1	3
**Rab35-B**	CTTGAAGTCGACTCCTATA	2	1	0	1

Mismatches identified by Cas OFFFinder.

Supplemental Figure 11. Rab35 alters RhoA activity in sprouts and CRISPR off-target sites in zebrafish. (A) Representative image of GFP-RhoA-Biosensor in scramble (Scram) and Rab35 siRNA (si) treated sprouts. L denotes lumen, white dashed line outlines sprouts and insets are higher magnification**. (B)** Table showing Rab35A/B single-guide RNA potential off-target genes with one or two nucleotide mismatches.

Reagent	Vendor	Catalog #
OPTI-MEM 1 Reduced Serum Medium, no phenol red	ThermoFisher	31985070
Polyethyleneamine Branched (PEI)	Sigma-Aldrich	408727
Chloroquine Diphosphate Crystalline (CQ)	Sigma-Aldrich	C6628-25G
Endothelial Cell Growth Medium 2	PromoCell	C-22011
DMEM, High Glucose, with L-Glutamine	Genesee Scientific	25-500
GenClone Fetal Bovine Serum (FBS)	Genesee Scientific	25-514
Penicillin-Streptomycin 100X Solution	Genesee Scientific	P4333-100ML
DPBS, no Calcium, no Magnesium	ThermoFisher	14190250
Trypsin-EDTA, o.25% 1X, phenol red	Genesee Scientific	25-510
Paraformaldahyde 20% Aqueous Sol. EM Grade	Electron Microscopy Sciences	15713
BSA Lyophilized Powder, Fraction V	Genesee Scientific	25-529
Cytoskeleton G actin/ F actin In Vivo Assay Kit	Cytoskeleton, Inc.	BK037-BK037
Culture-Insert 2 Well in µ-Dish 35	Ibidi	81176
Dimethyl Sulfoxide (DMSO)	Sigma-Aldrich	D2650-5X10ML
Silencer™ Negative Control No. 1 siRNA	ThermoFisher	AM4611
Rab35 siRNA	ThermoFisher	siRNA ID: s21709
ACAP2 siRNA	ThermoFisher	siRNA ID: s24011
OCRL siRNA	ThermoFisher	siRNA ID: s9819
MICAL-L1 siRNA	Thermo Scientific	siRNA ID: s39940

Rusc2 siRNA	Thermo Scientific	siRNA ID: s19070
Podxl siRNA	Thermo Scientific	siRNA ID: s10771
DENNd1a siRNA	Thermo Scientific	siRNA ID: s33637
DENNd1b siRNA	Thermo Scientific	siRNA ID: s29140
DENNd1c siRNA	Thermo Scientific	siRNA ID: s36719
MICAL-1 siRNA	Thermo Scientific	siRNA ID: s230028
Arf6 siRNA	Thermo Scientific	siRNA ID: s1565
Cytodex Microcarrier Beads	Sigma-Aldrich	C3275-10G
Trimethoprim (TMP)	Sigma-Aldrich	T7883-5G
High-Capacity Reverse Transcription Kit	ThermoFisher	4368814
Fibrinogen Type 1-S from Bovine Plasma	Sigma-Aldrich	F8630-1G
Thrombin from Bovine Plasma	Sigma-Aldrich	T7513-500UN
Aprotinin Protease Inhibitor	ThermoFisher	78432
Phenol-Red (Zebrafish Injection Mixture)	Avantor/ VWR	34487-61-1
CRIPSR gRNA	Integrated DNA Technologies (IDT)	
Alt-R® S.p. Cas9 Nuclease V3, 100 µg	Integrated DNA Technologies (IDT)	1081058
CellTracker Deep Red	ThermoFisher	M22426
3-Aminobenzoic Acid Ethyl Ester (Tricaine)	Sigma-Aldrich	A5040-25G
Latex Beads, Polystyrene Carboxylate Mod	Sigma-Aldrich	L3280-1ML
Dynabeads™ Protein G for Immunoprecipitation	ThermoFisher	10003D
MitoTracker DeepRed	ThermoFisher	M22426
Trizol Reagent	ThermoFisher	15596026
Chloroform	Sigma-Aldrich	288306

MEGAscript™ T3 Transcription Kit	ThermoFisher	AM1338
BCA Protein Assay Kit	ThermoFisher	23225
NHLF	Lonza	CC-2512
HEK 293-A	ThermoFisher	R70507
Microcarrier beads	Amersham	17-0485-01
Protease inhibitor cocktail	GoldBio	GB-334-20
Agarose Resin	GoldBio	G-250-G
Fura Red™, AM, cell permeant	ThermoFisher	F3020
Arf6 Pull-Down Activation Assay Biochem Kit	Cytoskeleton, Inc.	BK033
RhoA Pull-Down Activation Assay Biochem Kit	Cytoskeleton, Inc.	BK036
Rac1 Pull-Down Activation Assay Biochem Kit	Cytoskeleton, Inc.	BK035

Supplementary Table 1. Reagents.

**Name**	**Vendor or Source**	**Catalog No.**	**Working Concentration**
NP-G2-044	Selleck Chem	S2962	10mM
CN02	Cytoskeleton, Inc.	CN02-A	1unit/mL
CN03	Cytoskeleton, Inc.	CN03-A	5ug/ mL
NSC	Sigma-Aldrich	SML0952-5MG	100mM
Y-27632	Sigma-Aldrich	688001-500UG	10mM
CK-666	Sigma-Aldrich	SML0006-5MG	40mM

Supplementary Table 2. Drug treatment compounds.

**Target Antigen**	**Vendor or Source**	**Catalog No.**	**Working Concentration**
Rab35	ThermoFisher	PA531674	1:500 (WB)
ACAP2	ThermoFisher	PA557069	1:500 (WB)

OCRL	ThermoFisher	PA527844	1:200 (WB)
MICAL-L1	ThermoFisher	PA5107177	1:200 (WB)
Rusc2	ThermoFisher	PA572752	1:200 (WB)
Arf6	Santa Cruz	sc-7971	1:200 (WB)
Myc-tag	ThermoFisher	132500	1:1000 (IHC)
HA-tag	ThermoFisher	26183	1:1000 (IHC)
cyan	Bio-Rad	AHP2986	1:1000 (IHC)
Alpha-tubulin	Abcam	ab52866	0.0648ug/mL (1:10,000) (WB)
GAPDH	ThermoFisher	PA1988	1:1000 (WB)
Moesin	Abcam	ab52490	0.05ug/mL (1:1000) (IHC)
VE-Cadherin	ThermoFisher	14-1441-82	0.5ug/mL (1:1000) (IHC)
Podocalyxin	R&D	AF1658	15ug/mL (1:200) (WB & IHC)
Von Willebrand Factor	Abcam	ab6994	10ug/mL (1:1000) (IHC)
β1-Integrin	Abcam	ab30394	1:500 (IHC)
MICAL-1	ThermoFisher	14818-1-AP	1:500 (WB)
Phosphorylated TIE-2/TEK (Tyr992)	Sigma Aldrich	ABF131	0.25 ug/mL (1:500) (IHC)
Anti-HA-Tag, Rabbit Monoclonal	Sigma-Aldrich	SAB5600116-100UG	5ug/mL
Alexa Fluor™ 488 Phalloidin	ThermoFisher	A12379	1 uM (1:200)

Alexa Fluor™ 647 Phalloidin	ThermoFisher	A22287	1 uM (1:200)
Alexa Fluor™ 555 Phalloidin	ThermoFisher	A34055	1 uM (1:200)
Goat anti-Rabbit IgG (H+L) Secondary Antibody, Alexa Fluor 488	ThermoFisher	A11008	1ug/mL (1:500)
Donkey anti-Rabbit IgG (H+L) Secondary Antibody, Alexa Fluor 555	ThermoFisher	A31572	1ug/mL (1:500)
Donkey anti-goat IgG (H+L) Secondary Antibody, Alexa Flour 488	ThermoFisher	A11055	1ug/mL (1:500)
Donkey anti-Goat IgG (H+L) Cross-Adsorbed Secondary Antibody, Alexa Fluor 555	ThermoFisher	A21432	1ug/mL (1:500)
Chicken anti-Rabbit IgG (H+L) Cross-Adsorbed Secondary Antibody, Alexa Fluor 647	ThermoFisher	A21443	1ug/mL (1:500)
Goat Anti-Rabbit HRP	Genesee Scientific	20-303	1ug/mL (1:500)

Supplementary Table 3. Antibodies.

**Name**	**Vendor or Source**	**Catalog No.**
GFP-Rab35 WT	Addgene	47426
GFP_Rab35 Q67L active	Addgene	47425
GFP-Rab35 S22N inactive	Addgene	47426
pARF6-CFP	Addgene	11382
pARF6(T27N)-CFP	Addgene	11386

pARF6(Q67L)-CFP	Addgene	11387
pcDNA3-HA-human OCRL	Addgene	22207
mEmerald-Fascin-C-10	Addgene	54094
mEmerald-ARP2-C-14	Addgene	53992
mCherry-ARP2-N-14	Addgene	54980
pCDNA3.0_mitoLAMA-G97	Addgene	130705
pEGFP-RhoA Biosensor	Addgene	68026
MICAL-L1 (GFP-tagged) - Human MICAL-like 1 (MICAL-L1)	Origene	RG214051
DENND1C (NM_024898) Human Tagged ORF Clone	Origene	RC206410

Supplementary Table 4. Plasmids.

**Name**	**Sequence**	**Function**
Rab35a crRNA	CCATCGGTGTGGACTTCAAG	sgRNA Target
Rab35b crRNA	CTATAGGAGTCGACTTCAAG	sgRNA Target
Rab35a_seqF	GCCAATCAGATTCGAGATCCAGAC	Sequencing Primer
Rab35a_seqR	CACTCACGTGGAGGTGATTGTCCTG	Sequencing Primer
Rab35b_seqF	CACGCATAGTTCAATGGTGTGTG	Sequencing Primer
Rab35b_seqR	GCACACCCCTATCATGACACTACTC	Sequencing Primer

Supplementary Table 5. Genotyping primers and CRISPR sgRNA sequences.
