# Supplementary material for: Rab35 governs apicobasal polarity through regulation of actin dynamics during sprouting angiogenesis
Source: Nat Commun. 2022 Sep 8;13:5276. doi: 10.1038/s41467-022-32853-5 (PMC9458672; doi:10.1038/s41467-022-32853-5)
Supplement: Supplementary file 3 — Description of Additional Supplementary Files [file 41467_2022_32853_MOESM3_ESM.pdf]

Description of Additional Supplementary Files

File Name: Supplementary Movie 1
Description: Sprout expressing GFP-Rab35. L denotes lumen.

File Name: Supplementary Movie 2
Description: Sprout expressing GFP-Rab35 and LifeAct-TagRFP647 (FarRed) treated with scrambled siRNA. L denotes lumen.

File Name: Supplementary Movie 3
Description: Sprout expressing GFP-Rab35 and LifeAct-TagRFP647 (FarRed) treated with DENNd1c siRNA. L denotes lumen. Arrow marks actin accumulation.

File Name: Supplementary Movie 4
Description: Sprout expressing GFP-Rab35 and LifeAct-TagRFP647 (FarRed) treated with DMSO. Arrow marks normal actin buttressing at junctions.

File Name: Supplementary Movie 5
Description: Sprout expressing GFP-Rab35 and LifeAct-TagRFP647 (FarRed) treated with CK-666. Arrow marks actin accumulations.

File Name: Supplementary Movie 6
Description: Cell expressing TagRFP-Rab35 and GFP-LifeAct before and after CK-666 treatment.

File Name: Supplementary Movie 7
Description: Cell expressing TagRFP-DENNd1c and GFP-LifeAct before and after CK-666 treatment.

File Name: Supplementary Movie 8
Description: Cell expressing mCherry-Arp2 and GFP-Rab35 before and after CK-666 treatment.

File Name: Supplementary Movie 9
Description: Cell expressing GFP-Rab35, LifeAct-TagRFP647 (FarRed), and ligand-modulated antibody fragments targeted to the mitochondria (mito-LAMA) before and after trimethoprim (TMP) administration.

File Name: Supplementary Movie 10
Description: Cell expressing GFP-Rab35, mCherry-Arp2, and ligand-modulated antibody fragments targeted to the mitochondria (mito-LAMA) before and after trimethoprim (TMP) administration.

File Name: Supplementary Movie 11
Description: Cell expressing GFP-Rab35, TagRFP-DENNd1c, and ligand-modulated antibody fragments targeted to the mitochondria (mito-LAMA) before and after trimethoprim (TMP) administration.

File Name: Supplementary Movie 12
Description: Cell expressing GFP-Rab35, mCherry-Arp2, and ligand-modulated antibody fragments targeted to the mitochondria (mito-LAMA) before and after trimethoprim (TMP) administration. After TMP treatment cell were also treated with CK-666.

File Name: Supplementary Movie 13

Description: DENNd1c knockdown (siRNA) cell expressing GFP-Rab35, mCherry-Arp2, and ligand-modulated antibody fragments targeted to the mitochondria (mito-LAMA) before and after trimethoprim (TMP) administration.
