# Supplementary material for: Rab35 governs apicobasal polarity through regulation of actin dynamics during sprouting angiogenesis
Source: Nat Commun. 2022 Sep 8;13:5276. doi: 10.1038/s41467-022-32853-5 (PMC9458672; doi:10.1038/s41467-022-32853-5)
Supplement: Supplementary file 17 — Reporting Summary [file 41467_2022_32853_MOESM17_ESM.pdf]

Corresponding author(s): Erich J. Kushner

Last updated by author(s): 20222-07-29

Reporting Summary

Nature Portfolio wishes to improve the reproducibility of the work that we publish. This form provides structure for consistency and transparency in reporting. For further information on Nature Portfolio policies, see our Editorial Policies and the Editorial Policy Checklist.

Statistics

For all statistical analyses, confirm that the following items are present in the figure legend, table legend, main text, or Methods section.

n/a	Confirmed	
☐	☒	The exact sample size (*n*) for each experimental group/condition, given as a discrete number and unit of measurement
☐	☒	A statement on whether measurements were taken from distinct samples or whether the same sample was measured repeatedly
☐	☒	The statistical test(s) used AND whether they are one- or two-sided<br>*Only common tests should be described solely by name; describe more complex techniques in the Methods section.*
☒	☐	A description of all covariates tested
☒	☐	A description of any assumptions or corrections, such as tests of normality and adjustment for multiple comparisons
☐	☒	A full description of the statistical parameters including central tendency (e.g. means) or other basic estimates (e.g. regression coefficient) AND variation (e.g. standard deviation) or associated estimates of uncertainty (e.g. confidence intervals)
☐	☒	For null hypothesis testing, the test statistic (e.g. *F*, *t*, *r*) with confidence intervals, effect sizes, degrees of freedom and *P* value noted<br>*Give P values as exact values whenever suitable.*
☒	☐	For Bayesian analysis, information on the choice of priors and Markov chain Monte Carlo settings
☒	☐	For hierarchical and complex designs, identification of the appropriate level for tests and full reporting of outcomes
☐	☒	Estimates of effect sizes (e.g. Cohen's *d*, Pearson's *r*), indicating how they were calculated

Our web collection on statistics for biologists contains articles on many of the points above.

Software and code

Policy information about availability of computer code

Data collection	Microsoft Excel, APADT for quantification of cell dynamics (Barry et al. JCB. 2015)
Data analysis	Prism GraphPad

For manuscripts utilizing custom algorithms or software that are central to the research but not yet described in published literature, software must be made available to editors and reviewers. We strongly encourage code deposition in a community repository (e.g. GitHub). See the Nature Portfolio guidelines for submitting code & software for further information.

Data

Policy information about availability of data

All manuscripts must include a data availability statement. This statement should provide the following information, where applicable:

- Accession codes, unique identifiers, or web links for publicly available datasets
- A description of any restrictions on data availability
- For clinical datasets or third party data, please ensure that the statement adheres to our policy

All raw data is provided is the source data file accompanying the manuscript. Any additional data will be provided up request.

Human research participants

Policy information about studies involving human research participants and Sex and Gender in Research.

Reporting on sex and gender	NA
Population characteristics	NA
Recruitment	NA
Ethics oversight	NA

Note that full information on the approval of the study protocol must also be provided in the manuscript.

Field-specific reporting

Please select the one below that is the best fit for your research. If you are not sure, read the appropriate sections before making your selection.

☒ Life sciences ☐ Behavioural & social sciences ☐ Ecological, evolutionary & environmental sciences

For a reference copy of the document with all sections, see nature.com/documents/nr-reporting-summary-flat.pdf

Life sciences study design

All studies must disclose on these points even when the disclosure is negative.

Sample size	All sample sizes are
Data exclusions	no exclusions
Replication	A minimum of three replicate were performed for all experiments-all replicated data was included for analysis.
Randomization	Randomization was not performed. Covariate testing was not applicable to the study design.
Blinding	No blinding was performed. Was not relevant to study design primarily using cell culture assays.

Reporting for specific materials, systems and methods

We require information from authors about some types of materials, experimental systems and methods used in many studies. Here, indicate whether each material, system or method listed is relevant to your study. If you are not sure if a list item applies to your research, read the appropriate section before selecting a response.

Materials & experimental systems

n/a	Involved in the study	
☐	☒	Antibodies
☐	☒	Eukaryotic cell lines
☒	☐	Palaeontology and archaeology
☐	☒	Animals and other organisms
☒	☐	Clinical data
☒	☐	Dual use research of concern

Methods

n/a	Involved in the study	
☒	☐	ChIP-seq
☒	☐	Flow cytometry
☒	☐	MRI-based neuroimaging

Antibodies

Antibodies used

Rab35 ThermoFisher PA531674
ACAP2 ThermoFisher PA557069
OCRL ThermoFisher PA527844
MICAL-L1 ThermoFisher PA5107177
Rusc2 ThermoFisher PA572752
Arf6 Santa Cruz sc-7971
Myc-tag ThermoFisher 132500
HA-tag ThermoFisher 26183

cyan Bio-Rad AHP2986
Alpha-tubulin Abcam ab52866
GAPDH ThermoFisher PA1988
Moesin Abcam ab52490
VE-Cadherin ThermoFisher 14-1441-82
Podocalyxin R&D AF1658
Von Willebrand Factor Abcam ab6994
β1-Integrin Abcam ab30394
MICAL-1 ThermoFisher 14818-1-AP
Phosphorylated TIE-2/TEK Sigma Aldrich ABF131

Validation

All antibodies were purchased from noted commercial sources and validated by the manufacturer as mentioned on their websites. Additionally, all antibodies were validated for correct band size if used in western blot analysis or correct cellular localization if used for IHC.

Eukaryotic cell lines

Policy information about cell lines and Sex and Gender in Research

Cell line source(s)	Pool-Human umbilical vein endothelial cells (Promocell, C-12208)<br>Normal Human lung fibroblasts (ATCC, PCS-201-013)<br>HEK293A (ThermoFisher, R70507)
Authentication	Cells were validated for purity by vendor via antigen testing and morphology.
Mycoplasma contamination	Negative
Commonly misidentified lines<br>(See ICLAC register)	No commonly misidentified lines

Animals and other research organisms

Policy information about studies involving animals; ARRIVE guidelines recommended for reporting animal research, and Sex and Gender in Research

Laboratory animals	Zebrafish, Danio rerio, strain AB, used 24-72 hours post fertilization
Wild animals	NA
Reporting on sex	Sex not determined at time of use.
Field-collected samples	NA
Ethics oversight	All animal studies were approved by the University of Denver IACUC in accordance with AAALAC recommendations.

Note that full information on the approval of the study protocol must also be provided in the manuscript.
